# Catalytic Abatement of Volatile Organic Compounds and Soot over Manganese Oxide Catalysts

**DOI:** 10.3390/ma14164534

**Published:** 2021-08-12

**Authors:** Miguel Jose Marin Figueredo, Clarissa Cocuzza, Samir Bensaid, Debora Fino, Marco Piumetti, Nunzio Russo

**Affiliations:** Department of Applied Science and Technology, Politecnico di Torino, Corso Duca degli Abruzzi 24, 10129 Torino, Italy; miguel.marinfigueredo@polito.it (M.J.M.F.); clarissa.cocuzza@polito.it (C.C.); samir.bensaid@polito.it (S.B.); debora.fino@polito.it (D.F.); nunzio.russo@polito.it (N.R.)

**Keywords:** manganese oxide catalysts, soot catalytic oxidation, catalytic oxidation of volatile organic compounds, sustainable catalysts

## Abstract

A set of manganese oxide catalysts was synthesized via two preparation techniques: solution combustion synthesis (Mn_3_O_4_/Mn_2_O_3_-SCS and Mn_2_O_3_-SCS) and sol-gel synthesis (Mn_2_O_3_-SG550 and Mn_2_O_3_-SG650). The physicochemical properties of the catalysts were studied by means of N_2_-physisorption at −196 °C, X-ray powder diffraction, H_2_ temperature-programmed reduction (H_2_-TPR), soot-TPR, X-ray photoelectron spectroscopy (XPS) and field-emission scanning electron microscopy (FESEM). The high catalytic performance of the catalysts was verified in the oxidation of Volatile Organic Compounds (VOC) probe molecules (ethene and propene) and carbon soot in a temperature-programmed oxidation setup. The best catalytic performances in soot abatement were observed for the Mn_2_O_3_-SG550 and the Mn_3_O_4_/Mn_2_O_3_-SCS catalysts. The catalytic activity in VOC total oxidation was effectively correlated to the enhanced low-temperature reducibility of the catalysts and the abundant surface O_α_-species. Likewise, low-temperature oxidation of soot in tight contact occurred over the Mn_2_O_3_-SG550 catalyst and was attributed to high amounts of surface O_α_-species and better surface reducibility. For the soot oxidation in loose contact, the improved catalytic performance of the Mn_3_O_4_/Mn_2_O_3_-SCS catalyst was attributed to the beneficial effects of both the morphological structure that—like a filter—enhanced the capture of soot particles and to a probable high amount of surface acid-sites, which is characteristic of Mn_3_O_4_ catalysts.

## 1. Introduction

In recent years, the elevated concentration of pollutant substances in the air has increased the occurrence of respiratory diseases and deaths in Europe and around the world [1,2,3,4]. This encouraged the placement of regulations on the emission of PM and/or VOCs in several countries [5,6]. Volatile organic compounds are a set of substances produced every year either through natural processes or as the result of human activities (biogenic and anthropogenic, respectively) [7]. On the other hand, particulate matter is formed by elemental carbon aggregates with surface-adsorbed organic compounds, sulfur and metal oxides. It is formed due to the pyrolysis of hydrocarbons, concurrent with a defective, stoichiometric amount of oxygen, leading to the nucleation of solid particles [8,9].

In order to realize the goal of reducing emissions of these pollutants to acceptable amounts, different abatement technologies have been developed. Adsorption, thermal oxidation, catalytic oxidation, and other techniques have been used to diminish VOCs emission [10,11,12,13,14,15,16,17]. The elimination of carbonaceous particulate matter has been investigated and applied primarily in the automotive sector, e.g., in diesel particulate filters [18]. Consistently, the catalytic oxidation process has demonstrated its suitability for use in the elimination of VOCs and PM by means of active catalysts. 

Concerning the elimination of VOCs, many catalytic materials, e.g., noble metals and metal oxides, have been studied. The former materials are usually characterized by remarkable catalytic performance, but are more expensive and may be subject to poisoning [13,19]. On the other hand, metal oxides are low-cost materials but have been shown to be catalytically active at higher temperatures [10,13]. The enhanced catalytic activity of transition metal oxides, as reported by multiple research investigations, has been attributed to several factors, including: high amounts of surface-chemisorbed oxygen species and enhanced oxygen mobility (associated with the catalyst reducibility) [20,21], an electron-deficient lattice with enhanced conductivity (i.e., occurrence of positive holes), and the ability of the metal to assume variable oxidation states [22]. Manganese is among the most abundant elements in the Earth’s crust [23]; therefore, it constitutes a potential candidate material for the preparation of sustainable catalytic materials. Previous investigations demonstrated the outstanding catalytic performance of oxides containing manganese in different oxidation states (Mn_2_O_3_, Mn_3_O_4_ and MnO_x_) in the abatement of different VOCs like ethene, propene, and toluene [20]. The latter study confirmed the beneficial role played by surface-adsorbed electrophilic oxygen species and acidic sites in enhancing the catalytic activity.

In this work, a set of manganese oxide catalysts was synthesized via two different preparation techniques: solution combustion (SCS) and sol-gel (SG) syntheses. These procedures were used in the preparation of catalysts with different physicochemical and catalytic properties. The physicochemical properties of the prepared powder catalysts were studied by means of the following characterization techniques: N_2_-physisorption at −196 °C, X-ray powder diffraction, H_2_ temperature-programmed reduction, soot temperature-programmed reduction, X-ray photoelectron spectroscopy (XPS) and scanning electron microscopy (SEM). The high performance of the prepared catalysts was verified in the oxidation of VOC probe molecules (ethene and propene) and carbon soot.

## 2. Materials and Methods

### 2.1. Catalysts Preparation

The set of powder catalysts that were synthesized were composed of manganese oxide (MnO_x_). The catalysts were prepared by means of two preparation techniques: (i) solution combustion synthesis (SCS) and (ii) sol-gel (SG) synthesis. The different operative conditions applied in each procedure allowed for the preparation of powder catalysts with different physicochemical characteristics.

#### 2.1.1. Solution Combustion Synthesis

During the preparation of the Mn_2_O_3_ -SCS catalyst, an aqueous solution (50 mL) containing 0.2 mol·L^−1^ of the metal precursor, manganese nitrate tetrahydrate, (Mn(NO_3_)_2_•4H_2_O, Sigma-Aldrich), and 0.2 mol·L^−1^ of citric acid (CA, C_6_H_8_O_7_•H_2_O, Sigma-Aldrich) was prepared. The solution was magnetically stirred for 10 min until the powders were completely dissolved. Subsequently, the solution was heated in the oven from room temperature (r.t.) to 650 °C (5 °C·min^−1^), and calcined isothermally for 30 min. On the other hand, for the preparation of the Mn_3_O_4_/Mn_2_O_3_-SCS, a molar ratio of 1:1.25 between the Mn nitrate and CA was assured in the solution. After 10 min of stirring, the solution was heated from r.t. to 600 °C (10 °C·min^−1^) and calcined at a constant temperature for 30 min. Finally, the obtained powders were ground using mortar and pestle.

#### 2.1.2. Sol-Gel Synthesis

During the syntheses, manganese nitrate (0.2 mol·L^−1^) was dissolved with CA (0.2 mol·L^−1^) in 50 mL of Milli-Q water. After the powders were completely dissolved, the pH was increased (until pH = 5 was reached) by adding ammonium hydroxide (NH_4_OH, Sigma-Aldrich) dropwise. Afterward, the solution was set in a water bath and the temperature was raised (ca. 1 °C·min^−1^) to 60 °C under continuous stirring. The final temperature was maintained constantly for 2 h to enhance the nucleation and aggregation of colloidal particles. Afterward, the particles were separated from the suspension by means of vacuum filtration and washed with water. The resulting solid was dried at 60 °C overnight. Subsequently, it was heated (5 °C·min^−1^) in an oven from r.t. to (i) 550 °C (for the Mn_2_O_3_-SG550) and (ii) 650 °C (for the Mn_2_O_3_-SG650), respectively. The resulting catalyst was gently ground using mortar and pestle.

### 2.2. Catalysts Characterization

X-ray powder diffraction (XRD) analyses were performed by means of an X’Pert Philips PW3040 diffractometer. During measurements, Cu K_α_ radiation was utilized and the 2θ was varied in the 20° to 80° range; step = 0.05° 2θ and time per step = 0.2 s. The diffraction patterns were indexed according to the Powder Data File (PDF 2000, International Centre of Diffraction Data) Database. The average size of the crystallites was estimated using the Scherrer formula, D=0.9λ/bcosθ, where *λ* is the Cu K_α_ radiation wavelength, 0.9 the shape factor for spherical particles, *b* the full width at half maximum (FWHM) in radians, and θ the angle corresponding to the observed diffraction peak.

The specific surface area (S_BET_), total pore volume and pore diameter were calculated by the performance of N_2_ physisorption at −196 °C (Micromeritics Tristar II 3020). Before carrying out the analysis, the powders were pretreated under a N_2_ flow 200 °C for 2 h. The S_BET_ were estimated by means of the Brunauer–Emmett–Teller (BET) method. The diameter and volume of the pores (D_p_ and V_p_, respectively) were calculated using the method of Barrett–Joyner–Halenda (BJH) in the data corresponding to the desorption phase.

The morphology of the powder catalysts was observed by means of a field emission scanning electron microscope (FESEM) FEI QUANTA 200F, using the following operation parameters: HV = 10.00 kV, working distance = 4–5.4 mm, intensity = 46–180 nA. 

The reducibility of the catalysts was investigated through the realization of H_2_ temperature-programmed reduction (H_2_-TPR) in a ThermoQuest TPD/R/O 1100 analyzer. For the analyses, a thermal conductivity detector (TCD) was utilized. Prior to the performance of the analysis, the studied sample (ca. 20 mg) was subject to pretreatment under an He flow (40 mL·min^−1^) at 550 °C for 60 min. Subsequently, the temperature was lowered to r.t. and the analysis was undertaken. During the analysis phase, the sample was reduced under a flow of 5 vol.%-H_2_ in Ar (20 mL·min^−1^) and the temperature was increased at a constant rate (5 °C·min^−1^) from r.t. to 800 °C.

The soot-TPR technique was used to analyze the reducibility of the catalyst by soot. The technique was performed inside a quartz U-tube reactor (internal diameter ID = 4 mm), comprising a fixed-bed containing 45 mg of catalyst, 5 mg of soot (Printex-U), and 150 mg of silica in tight contact. During the analysis, the temperature of the reactor was increased by means of a PID-controlled furnace. The intimate soot-catalyst contact condition (i.e., tight contact) was assured in a ball-milling apparatus operating at 250 rpm for 15 min. Before the reduction, the powder mix was pretreated under N_2_ (100 mL·min^−1^) at 100 °C for 30 min. Afterward, the soot-TPR was carried out under flowing N_2_ (100 mL·min^−1^) while the temperature was increased to 700 °C (5 °C·min^−1^). The concentrations of CO_2_ and CO in the reactor outlet were estimated by means of NDIR analyzers.

X-ray photoelectron spectroscopy (XPS) investigations were conducted using an XPS PHI 5000 versa probe apparatus. The conditions applied during the analysis were as follows: band-pass energy = 187.85 eV, X-ray spot size = 100 μm, and take-off angle = 45°. The spectra obtained were fitted using Multipack 9.0.

### 2.3. Catalytic Activity Tests

The catalytic activity of the powders was investigated using a typical temperature-programmed oxidation setup. For the test, a fixed-bed containing the pelletized catalyst (diameter of pellets: 212–300 µm) was set in a quartz U-tube reactor (ID = 4 mm) and the temperature of the reactor was raised using a PID-controlled furnace. The temperature of the catalytic bed was estimated with a K-type thermocouple. The reactor outlet concentrations of CO and CO_2_ were measured by non-dispersive infrared (NDIR) analyzers (ABB Uras 14.

#### 2.3.1. Total Oxidation of VOC

Before the catalytic test, the catalyst (100 mg) was subjected to a degassing pretreatment under a flow of N_2_ at 150 °C for 1 h. The gaseous mixture for the test was prepared by diluting the VOC (propene or ethene) in air in order to obtain a reactor inlet concentration of 500 ppm-VOC, 10 vol.%-O_2_ and the balance in N_2_. The flow fed to the reactor was calculated according to a constant gas hourly space velocity (GHSV) of 20,000 h^−1^, equivalent to a weight-to-volumetric flowrate ratio (W/F) of 0.044 g h L^−1^. The isothermal steps (every 30 °C) performed during the analysis started at 70 °C, and the temperature was increased when a stable VOC conversion was observed (in terms of CO and CO_2_ concentrations).

#### 2.3.2. Oxidation of Soot

For the catalytic tests performed in loose contact between the catalyst and soot, 45 mg of catalyst, 5 mg of soot, and 150 mg of inert silica were gently mixed with a spatula for 3 min. The tight contact catalyst–soot condition was also obtained (as described previously) for the soot-TPR characterization procedure. The fixed-bed was pretreated under a flow of N_2_ (100 mL·min^−1^) at 100 °C for 30 min. Then, the catalytic test was carried out under gaseous flow (100 mL·min^−1^) containing 10 vol.% of O_2_ and the balance in N_2_. The temperature in the reactor was increased at a constant heating rate (5 °C·min^−1^) from 100 °C to 700 °C.

## 3. Results and Discussion

### 3.1. Material Textural Properties

**Figure 1:**
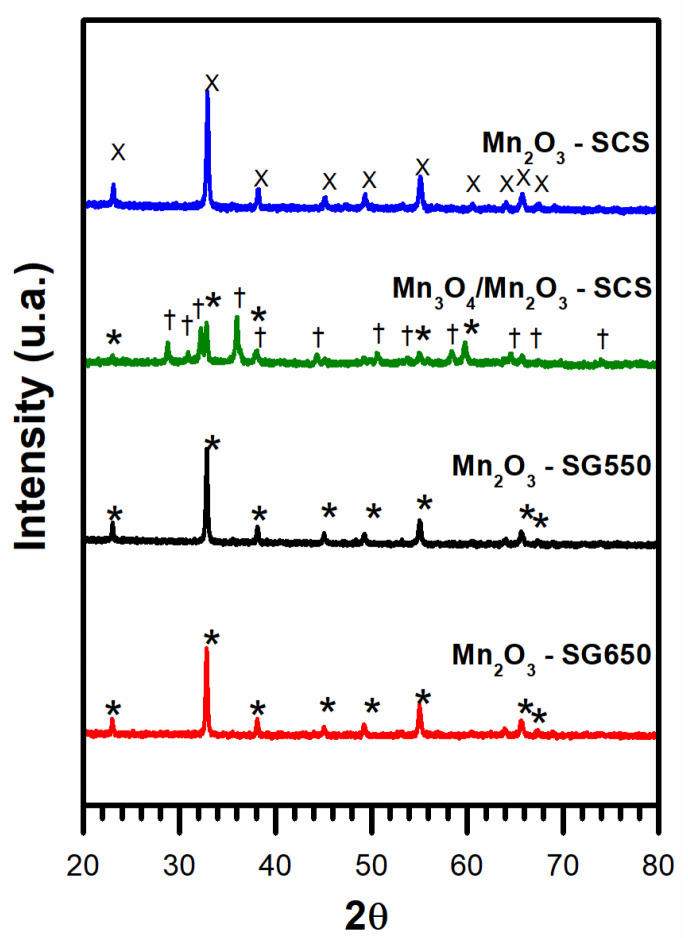
X-ray powder diffraction patterns of the synthesized catalysts. Assignments: Mn_2_O_3_ peaks = X and *; Mn_3_O_4_ = †.

In order to examine the crystalline phases present in the powder catalysts and their characteristics, XRD analyses were conducted. The diffraction patterns harvested during this technique are reported in Figure 1.

The patterns observed for the Mn_2_O_3_-SG550 and Mn_2_O_3_-SG650 consisted in the cubic structure of Mn_2_O_3_ (reference code 01-078-0390). This demonstrated that, in spite of the different calcination temperatures used after the sol-gel preparation, the resulting crystalline phase consisted in the system of Mn_2_O_3_. On the other hand, the pattern observed for the Mn_2_O_3_-SCS demonstrated the formation of the orthorhombic system of Mn_2_O_3_ (ref. code 01-073-1826). The Mn_3_O_4_/Mn_2_O_3_ catalyst showed a mixed diffraction pattern, mainly composed of hausmannite (ref. code 00-024-0734). However, additional diffraction lines appeared and were ascribed to the presence of Mn_2_O_3_ (ref. code 01-078-0390).

The textural properties of the samples are summarized in Table 1. The results show that the SCS procedure allowed us to obtain higher specific surface areas (S_BET_) with respect to the SG synthesis. Obviously, obtaining higher specific surface areas is a positive outcome, since it may improve the catalytic performance in gas-phase reactions due to the higher amount of available active sites. On the other hand, larger crystallites were obtained in the samples prepared via the SG preparation than in those obtained with the SCS technique.

**Figure 2:**
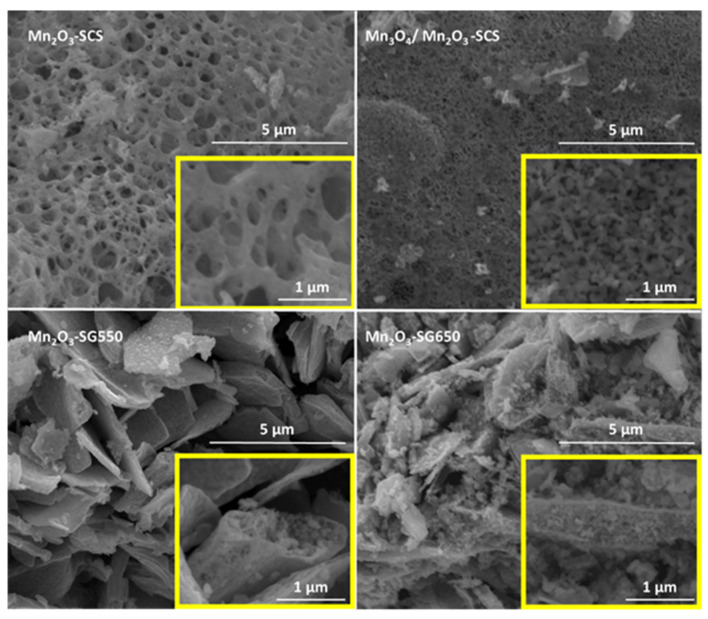
FESEM micrographs of the prepared catalysts and the corresponding magnifications (in the yellow frames).

The morphologies of the catalyst as observed in the FESEM are summarized in Figure 2. In general, the micrographs show the formation of mesoporous structures during both synthesis procedures. Moreover, the catalysts prepared via SCS were characterized by a mesoporous structure. This coincided with the highest surface areas observed, suggesting the formation of highly porous, sponge-like structures during the SCS technique. Conversely, the powders prepared via SG synthesis were formed by slim nanoplates. Interestingly, the micrographs showed that the nanoplates formed in the sol-gel synthesis contained a porous interior enclosed in an external shell. 

### 3.2. Temperature-Programmed Analyses

**Figure 3:**
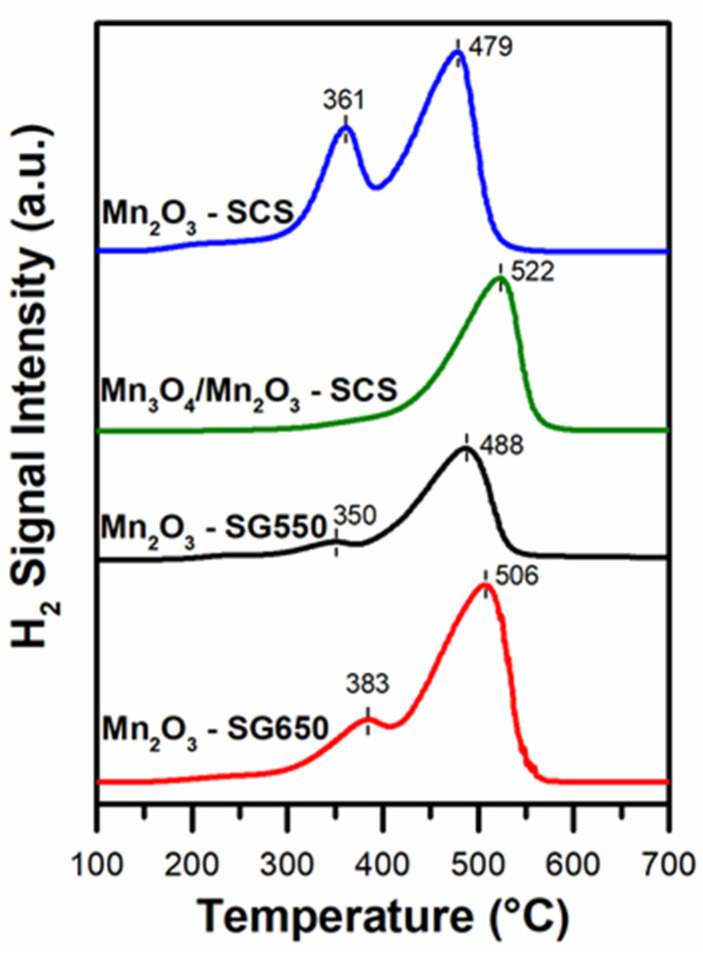
H_2_-TPR of the synthesized catalysts.

The reducibility of the catalysts was estimated under a flow of 5 vol.%-H_2_ in Ar in the range 100–800 °C. The reduction profiles obtained are shown in Figure 3. As a general behavior, the samples composed by Mn_2_O_3_ showed a two-peak reduction profile. Therefore, in this case, the reduction signal with maxima between 350–383 °C could be ascribed to the reduction step Mn_2_O_3_ → Mn_3_O_4_. Meanwhile, the peak which occurred at higher temperatures corresponded to the final reduction step Mn_3_O_4_ → MnO [20,24,25,26]. Consistently, this result confirmed the elevated amount of Mn species with oxidation states 3+ in samples composed by Mn_2_O_3_ (as observed during XRD analyses). Additionally, the formation of larger particles during SG synthesis (as estimated by means of Scherrer formula) seemed related to a decreased intensity of the first reduction peak (i.e., the low-temperature peak) and the translation of the second signal to higher temperatures. In accordance with the literature, this could be associated with the formation of larger particles of Mn_2_O_3_ which could be more resistant to reduction [27,28].

On the other hand, the small reduction signal of the Mn_3_O_4_/Mn_2_O_3_-SCS which began around 300 °C may be attributed to the presence of Mn^3+^ species in Mn_2_O_3_ crystals, as confirmed in the XRD studies (vide supra). On the whole, a reducibility trend (in terms of low-temperature reduction signal) was drawn as follows: Mn_2_O_3_-SG550 > Mn_2_O_3_-SCS > Mn_2_O_3_-SG650 > Mn_3_O_4_/Mn_2_O_3_-SCS.

**Figure 4:**
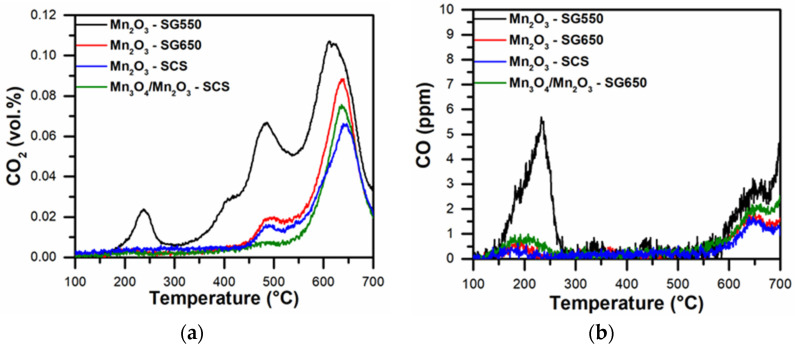
(**a**) CO_2_ and (**b**) CO profiles observed during the soot-TPR analyses of the prepared catalysts.

The reducibility of the catalysts by soot in tight contact condition was studied in the absence of oxygen (see Figure 4). Under this condition, the oxidation of carbonaceous matter took place by means of the oxygen species composing the catalyst. Various signal maxima of CO_2_ appeared in the whole range of temperatures analyzed. The first signal (observed only for the Mn_2_O_3_-SG550) appeared around 240 °C and was attributed to the desorption of CO_2_ bonded to basic sites of manganese oxide [29]. On the other hand, the signal observed at T < 450 °C was attributed to the oxidation of soot (C + O_α_ → CO + O_α_ → CO_2_) by means of chemisorbed O^2−^, while the signal that occurred between 450 and 550 °C was associated to other electrophilic oxygen species (e.g., O^−^ and O_2_^2−^) [30,31]. At higher temperatures, the oxidation of soot occurred as a result of the release of oxygen present in the bulk (C + O_β_ → CO + CO_2_), coinciding with the appearance of CO (see Figure 4b). The latter took place due to the slow diffusion of oxygen from the bulk to the surface, under which condition some of the reaction intermediate (thus the CO) did not convert to CO_2_ [32]. It is worth noting the lower signal intensity in the case of the catalysts calcined at 650 °C with respect to the signal of the Mn_2_O_3_-SG550. This result suggested that the higher calcination temperature may have stabilized the crystalline structure of the catalysts, thus leading to an overall lower reducibility. Consistently, the overall reducibility of the catalysts by soot followed this trend: Mn_2_O_3_-SG550 > Mn_2_O_3_-SG650 ≈ Mn_2_O_3_-SCS ≈ Mn_3_O_4_/Mn_2_O_3_-SCS.

### 3.3. X-ray Photoelectron Spectroscopy

**Figure 5:**
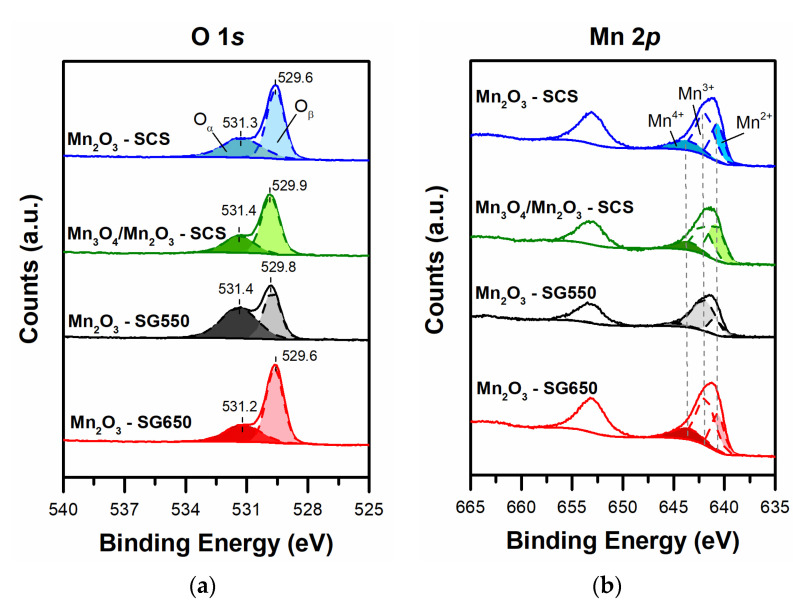
XPS spectra in the (**a**) O 1*s* (section a) and (**b**) Mn 2p core level.

The spectra obtained during the XPS investigations are summarized in Figure 5. The spectra obtained in the O 1*s* core level and the respective deconvolution are shown in Figure 5a. As a whole, the spectra were characterized by a signal with two maxima at different binding energy (BE) values, each one assigned to different oxygen species. The signals that appeared at low binding energy (between 529.6 and 529.9 eV) were attributed to nucleophilic lattice oxygen (O^2−^) bonded to Mn (thus O_β_ species) [33]. On the other hand, the signal maxima centered between 531.2 and 531.4 eV was ascribed to electrophilic oxygen species (e.g., O^−^ or O^2−^) or OH groups located over the surface of the catalyst [21,34]. The amount of O_α_ and O_β_ species (see Table 2) were estimated according to the deconvolution of the spectra. A decreasing trend of the O_α_/O_β_ ratio was observed as follows: Mn_2_O_3_-SG550 > Mn_2_O_3_-SCS > Mn_2_O_3_-SG650 ≈ Mn_3_O_4_/Mn_2_O_3_-SCS. Remarkably, the increased amount of O_α_ has been correlated in the literature to total oxidation of hydrocarbons [20,35,36].

Additionally, the XP spectra in the Mn 2p core level were measured, and are included in Figure 5b. The signal observed at higher binding energy was attributed to the 2p_1/2_ level, whereas the signal located at lower binding energy (between 637.8–647.4 eV) corresponded to the 2p_3/2_ level. As reported in the literature, the deconvolution of the latter signal can be used for estimating the relative amount of Mn^x+^ species (where x = 2+; 3+ and 4+) in the sample [20,21,37]. The spectra deconvolution (numerical data not reported for the sake of brevity) confirmed the predominance of Mn species with oxidation state 3+ for the samples composed mainly by Mn_2_O_3_. On the other hand, the calculations on the amount of Mn^3+^ and Mn^2+^, as expected, revealed similar amounts as the sample composed by the spinel Mn_3_O_4_.

### 3.4. Catalytic Activity

#### 3.4.1. Oxidation of VOCs

**Figure 6:**
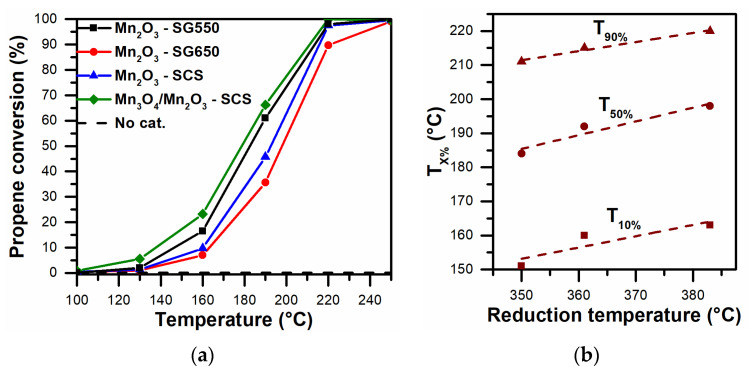
(**a**) Catalytic performances in the oxidation of C_3_H_6_ and (**b**) correlation between the low-temperature reduction peak and the catalytic performance in C_3_H_6_ oxidation (over Mn_2_O_3_ catalysts) in terms of T_10%_, T_50%_ and T_90%_.

**Figure 7:**
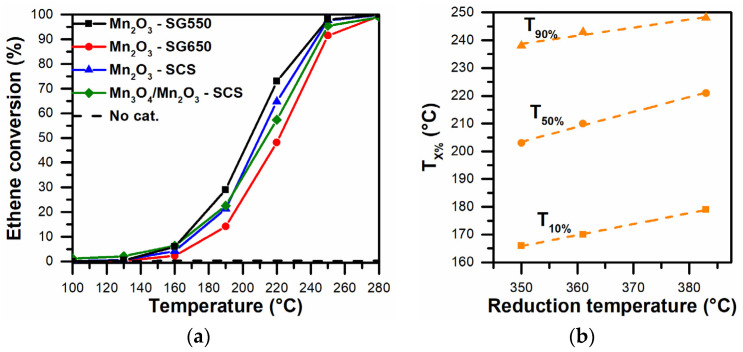
(**a**) Catalytic performances in the oxidation of C_2_H_4_ and (**b**) correlation between the low-temperature reduction peak and the catalytic performance in C_2_H_4_ oxidation (over Mn_2_O_3_ catalysts) in terms of T_10%_, T_50%_ and T_90%_.

The catalytic performance of the prepared catalysts was evaluated in the oxidation of carbon soot precursors (i.e., volatile organic compounds). During the testing of the samples, propene and ethene were used as probe VOC molecules. The catalytic performances observed during the tests are summarized in Figure 6 and Figure 7a. The whole set of catalysts completely converted the VOCs at lower temperatures, with respect to the blank test that had no catalyst. The VOC conversion rates of the prepared catalysts are summarized in Table 3.

The following trend can be outlined for the catalytic activity during the oxidation of propene: Mn_3_O_4_/Mn_2_O_3_-SCS > Mn_2_O_3_-SG550 > Mn_2_O_3_-SCS > Mn_2_O_3_-SG650. The general performances of the catalysts prepared via SG showed catalytic improvement when a lower calcination temperature was utilized. In fact, the higher temperature may have caused the sintering of the catalyst, leading to the reduction of the catalyst’s surface area (as reported in Table 1). Therefore, this finding confirms the key role of calcination temperature in the preparation of catalysts active in the oxidation of VOCs. The most active catalyst in the oxidation of propene was composed mainly of the spinel structure Mn_3_O_4_ (coupled Mn^3+^|Mn^2+^ species) with the smallest crystallite size of the prepared set of catalysts (approximately 37 nm for the Mn_3_O_4_). In this sense, the outstanding activity of the Mn_3_O_4_/Mn_2_O_3_-SCS catalyst could have been associated with the presence of small crystallites of hausmannite that, according to the literature, can promote an elevated occurrence of crystallite edges and corners and thus, more defective structures active in the oxidation reaction [38,39]. It is worth noting that, among the catalysts classified as pure Mn_2_O_3_ (by means of XRD), the catalytic trend coincided with a decreasing trend in two parameters: (i) low-temperature reducibility, and (ii) the amount of surface-chemisorbed O_α_-species. In fact, the catalytic performance data were fitted and showed a linear behavior dependent on the low-temperature reduction peak (see Figure 6b), thus confirming the correlation between reducibility and improved catalytic activity in propene oxidation (in terms of T_10%_, T_50%_ and T_90%_, thus the temperatures for achieving a conversion of 10%, 50% and 90%, respectively).

The Mn_2_O_3_-SG550 catalyst—in spite of having an intermediate surface area value with respect to the whole set of catalysts—showed the best overall catalytic performance in the abatement of ethene (see Figure 7a). Considering the overall catalytic performance and the low-temperature ethene reaction rates, the following catalytic activity trend in the oxidation of ethene was drawn: Mn_2_O_3_-SG550 > Mn_3_O_4_/Mn_2_O_3_-SCS > Mn_2_O_3_-SCS > Mn_2_O_3_-SG650. As a whole, the catalytic activity seemed to be attributable to the relative amount of active chemisorbed O_α_-species (as verified via XPS, vide supra) and (in the case of the Mn_2_O_3_ catalysts) the low-temperature reducibility of the catalysts (see Figure 7b).

Accordingly, the results demonstrate the key role played by active O_α_-species and catalyst reducibility in the elimination of ethene. In fact, according to the literature, a number of these species may promote VOC catalytic oxidation at lower temperatures in conjunction with low-temperature reducibility [20,21,27,40]. Overlooking the catalytic behavior observed during the abatement of the VOCs, it was evident that catalysts that belong to the same group or family could present different catalytic performances according to the abated molecule. In other words, the catalytic activity for a specific reaction may depend on a wide set of physicochemical properties (reducibility, surface acidity, size of crystallites, amount of chemisorbed oxygen species, etc.). Consequently, it may be useful to speculatively select a catalyst for a specific reaction according to a single physicochemical property or catalytic results obtained with other molecules. In conclusion, the prepared catalysts showed high levels of catalytic activity at relatively low temperatures in the elimination of VOCs (i.e., carbon soot precursors).

#### 3.4.2. Oxidation of Carbonaceous Matter (Soot)

**Figure 8:**
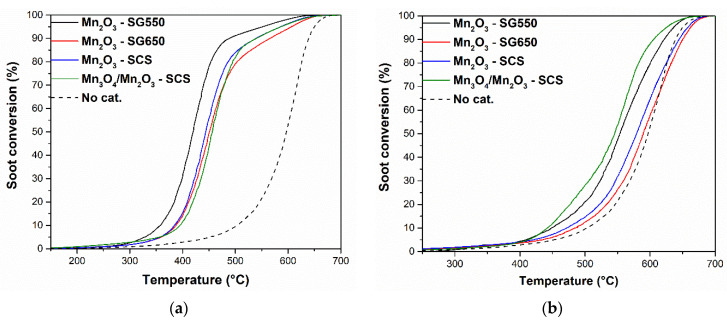
Catalytic conversion of carbon soot as a function of the temperature in (**a**) “tight” and (**b**) “loose” contact conditions.

The catalytic activity of the oxides was tested in the oxidation of carbonaceous matter in loose or tight contact. The former condition was assured by a delicate mixing of the powders (soot, catalyst, inert silica), whereas the most intimate solid–solid interactions (i.e., tight contact) were enhanced by means of the ball-milling process. The curves of the soot catalytic conversions of the prepared catalysts are included in Figure 8.

During all catalytic tests, CO_2_ was the main oxidation product, while the concentration of CO remained low (or even imperceptible) in most cases. The soot oxidation was a solid–solid reaction; therefore, enhancing catalyst–soot contact played a key role in estimations of soot oxidation kinetics [41]. In this sense, the conversions obtained in tight condition showed the intrinsic activity of the catalytic surface. Consistently, the results obtained in tight contact demonstrated that the catalytic performance of the active phase in the catalysts calcined at higher temperatures (T > 600 °C; see the blue, red, and green lines) are rather similar. On the other hand, the best performance was observed in the catalyst calcined at the lowest temperature, approximately 550°C (i.e., the Mn_2_O_3_-SG550). This catalyst achieved 10% and 50% of the soot conversion, respectively, at temperatures of approximately 25 °C below the other catalysts. These results suggested that the higher calcination temperatures may have enhanced a sintering process that diminished the whole number of soot–MnO_x_ contact points present in the catalysts. On the other hand, the reducibility of the catalysts was diminished in the catalysts calcined at higher temperatures (as demonstrated by soot–TPR analyses, vide supra), which also correlated with their lower catalytic performance in tight contact conditions. Furthermore, the best catalyst contained the highest amount of active O_α_-species, which are important for starting soot oxidation [18]. The results, therefore, demonstrate the remarkable importance of an elevated amount of surface O_α_ species and improved catalyst reducibility in the carbon soot oxidation process.

The loose contact conditions were more representative of real contact conditions in catalytic traps [41]. The results showed similar catalytic activities for the Mn_2_O_3_-SG650 and the Mn_2_O_3_-SCS. However, under this contact condition, the soot conversion of the catalyst containing the spinel Mn_3_O_4_ overcame the catalytic performance of the Mn_2_O_3_-SG550. This suggested that, in spite of the higher temperature treatment of the former catalyst, the contact points between soot and Mn_3_O_4_ greatly enhanced the catalytic performance. Accordingly, this further suggests that the contact points were readily accessible for the soot without favoring an intimate soot–catalyst contact. This could be associated to the sponge-like morphology of the catalyst, characteristic of the SCS preparation technique. This morphology resembled a catalytic trap or a filter. In this case, it may have allowed an easier path for soot to penetrate the structure of the catalyst and remain trapped inside it, with respect to the void nanoplates. Accordingly, this may have also enhanced the occurrence of contact points for the catalytic oxidation of soot. Moreover, in previous studies, Mn_3_O_4_ (and mixed oxides containing Mn_3_O_4_) were demonstrated to host an increased amount of acid sites with respect to other MnO_x_ [20,42]. Lewis acid sites are characterized by superficial (oxygen deficient) metal cations. These sites can play a key role in the adsorption of the oxygen needed for the oxidation reaction, its surface diffusion, and the final transfer to soot, Therefore, these sites can be correlated to soot oxidation performance [43,44]. Consistently, the elevated activity in loose catalyst–soot contact was ascribed to the combined promoting effects of the sponge-type morphology (acting like a powder filter or trap) and a probable increased amount of acid sites in contact with the soot of the Mn_3_O_4_/Mn_2_O_3_-SCS catalyst. The overall results demonstrated the remarkable effectiveness of sustainable MnO_x_-based oxides in the elimination of solid carbon soot and its organic precursors, VOCs.

## 4. Conclusions

A set of manganese oxide catalysts was synthesized via two preparation techniques: solution combustion synthesis (Mn_3_O_4_/Mn_2_O_3_-SCS and Mn_2_O_3_-SCS) and sol-gel synthesis (Mn_2_O_3_-SG550 and Mn_2_O_3_-SG650). These synthesis procedures allowed for the preparation of catalysts with different physicochemical and catalytic properties. Overall, the best catalytic performances in the abatement of solid carbon soot and VOCs were observed in Mn_2_O_3_-SG550 and Mn_3_O_4_/Mn_2_O_3_-SCS catalysts. Concerning the catalytic oxidation of the studied VOCs, the best catalytic performances were associated with the following parameters:

1. The elevated relative amounts of active surface O_α_ species, which can significantly improve the total catalytic oxidation of hydrocarbons at low temperatures.
2. The improved low-temperature reducibility of the catalysts due to the enhanced mobility of the oxygen taking part in the oxidation reactions.
3. The appearance of small crystallites, which may contain an elevated amount of surface defects that enhance the catalytic performance.

The influence of the aforementioned characteristics (i and ii) on the oxidative performance of the catalysts in the solid carbon soot oxidation reaction was demonstrated in the present study. Accordingly, in tight contact conditions, the best catalytic performance in soot oxidation was observed in the catalyst containing the highest amount of O_α_ species and exhibiting better reducibility, i.e., the Mn_2_O_3_-SG550. This result highlighted the beneficial effect of those two parameters on the intrinsic oxidative activity of the catalysts’ surface. On the other hand, the outstanding catalytic activity of the Mn_3_O_4_/Mn_2_O_3_-SCS in loose contact was attributed to the combined effect of (i) a filter-like morphology (that may have improved the capture of soot particles) and (ii) a probable high amount of surface acid sites, which are characteristic of Mn_3_O_4_ catalysts.

## Figures and Tables

**Table 1:** Textural properties of the synthesized catalysts, calculated by means of the N_2_ physisorption at −196 °C technique ^a^ and the Scherrer formula ^b^. S_BET_ = specific surface area (m^2^ g^−1^); Vp = Total pore volume (cm^3^ g^−1^); D_p_ = average pore diameter (nm); Crystallites size (nm).

Catalyst.	S_BET_^a^	V_P_^a^	D_P_^a^	Crystallites Size ^b^
Mn_2_O_3_-SG550	15	0.12	32	67
Mn_2_O_3_-SG650	11	0.10	37	61
Mn_2_O_3_-SCS	22	0.15	26	52
Mn_3_O_4_/Mn_2_O_3_-SG550	21	0.13	23	37/53

**Table 2:** Relative percentages (at.%) of oxygen species calculated from the deconvolution of the O 1*s* XPS spectra.

Catalyst	O_α_, OH^−^ BE (eV)	O_α_ (at.%)	O_β_ BE (eV)	O_β_ (at.%)	O_α_/O_β_
Mn_2_O_3_-SG550	531.4	56.7	529.8	43.3	1.31
Mn_2_O_3_-SG650	531.2	31.8	529.6	68.2	0.47
Mn_2_O_3_-SCS	531.3	38.5	529.6	61.5	0.63
Mn_3_O_4_/Mn_2_O_3_-SG550	531.4	31.8	529.9	68.2	0.47

**Table 3:** Propene and ethene specific reaction rates over the prepared catalysts.

Catalyst	r_propene_^a^ (μmol h^−1^ m^−2^)	r_ethene_^b^ (μmol h^−1^ m^−2^)
Mn_2_O_3_-SG550	0.94	1.67
Mn_2_O_3_-SG650	0.49	1.13
Mn_2_O_3_-SCS	0.35	1.04
Mn_3_O_4_/Mn_2_O_3_-SG550	1.48	1.69

^a^ calculated at 130 °C; ^b^ calculated at 160 °C.

## Data Availability

Not applicable.
